# Training Management of the Elite Adolescent Soccer Player throughout Maturation

**DOI:** 10.3390/sports9120170

**Published:** 2021-12-17

**Authors:** Alistair J. McBurnie, Thomas Dos’Santos, David Johnson, Edward Leng

**Affiliations:** 1 Football Medicine & Sports Science, Manchester United F.C., AON Training Complex, Manchester M31 4BH, UK; ed.leng@manutd.co.uk; 2 Department of Sport Science, School of Science and Technology, Nottingham Trent University, Nottingham NG1 4FQ, UK; 3 Musculoskeletal Science and Sports Medicine Research Centre, Department of Sport and Exercise Sciences, Manchester Metropolitan University, Manchester M15 6BH, UK; T.DosSantos@mmu.ac.uk; 4 Manchester Institute of Sport, Manchester Metropolitan University, Manchester M1 7EL, UK; 5 Department for Health, University of Bath, Bath BA2 7AY, UK; dmj32@bath.ac.uk

**Keywords:** long-term athlete development, soccer, growth and maturation, performance monitoring, injury surveillance

## Abstract

Professional soccer clubs invest significantly into the development of their academy prospects with the hopes of producing elite players. Talented youngsters in elite development systems are exposed to high amounts of sports-specific practise with the aims of developing the foundational skills underpinning the capabilities needed to excel in the game. Yet large disparities in maturation status, growth-related issues, and highly-specialised sport practise predisposes these elite youth soccer players to an increased injury risk. However, practitioners may scaffold a performance monitoring and injury surveillance framework over an academy to facilitate data-informed training decisions that may not only mitigate this inherent injury risk, but also enhance athletic performance. Constant communication between members of the multi-disciplinary team enables context to build around an individual’s training status and risk profile, and ensures that a progressive, varied, and bespoke training programme is provided at all stages of development to maximise athletic potential.

## 1. Introduction

Professional soccer clubs invest significant amounts of time and money into the development of academy prospects with the hopes of producing elite players (e.g., individuals formally registered to a professional soccer club) [1,2]. Popularity, competition, and new codes of conduct have contributed to a new scientific approach to the development of the academy soccer player, delivered by multi-disciplinary teams focussed on optimising training strategy. This approach aims to improve training methods and monitoring systems within developmental programmes to enhance long-term athletic development (LTAD), mitigate the relative risk of injury, and subsequently, improve chances of progression into the professional game to compete in their first team or at another club [3,4,5,6]. Increased investment into evidence-based talent identification and development models often apply systematic training in a single sport from a young age in an attempt to accumulate training exposure and practise [1,7,8,9]. Talent development models in soccer often create pathways of ‘early specialisation’ for young athletes, whereby early age involvement in one chosen sport (typically early- to middle-childhood), is partaken at the expense of participation in other sports or activities. In the United Kingdom (UK), the adoption of the Elite Player Performance Plan (EPPP) by elite soccer organisations is an example of such a model [10]. The EPPP was introduced by the English Premier League in 2011, aiming to provide an increased volume of on-pitch weekly training hours for talented youth soccer players [10]. The number of required contact hours for coaching has increased to 8500 h for clubs in the highest academy classification category, in contrast to the original 3760 h required in the original UK academy system set out in 1998. Furthermore, the linear approach adopted by the EPPP will mean that training exposure for individuals participating in these programmes may systematically increase by 20–50% as they progress through the age groups (from the age of 5 through to 16) [10,11].

There still remains much debate as to whether early-specialisation versus early-diversification practises are more effective training methods [12,13]. Researchers have provided evidence for the positive effects a high exposure to technical and tactical skill development from an early age has on fitness and motor control [13], which may thus provide a physical and technical foundation for the sports-specific capabilities needed in the game [14,15]. However, these types of talent development structures may expose the youth soccer player to an increased risk of injury [9,16,17,18]. This can not only be catastrophic in the sense of withdrawal from training and competition, but also means the end of participation in physical activity and sport for the youth athlete that may lead to long-term detrimental outcomes [19]. This increase in injury risk may be in part attributed to an increase in musculoskeletal system demands without allowing sufficient time for recovery and adaptation [16]. Chronic overuse injuries can account for up to 40% of all injuries in youth athletes and 20% of which can typically be classified as severe (i.e., an injury absence from sport for 4 weeks or more) [20]. Resultantly, a conflict may exist whereby the accumulated training hours necessary to exceed at the highest level (i.e., skill development) may come ‘hand-in-hand’ with an increased injury risk. Indeed, it has been shown that youth soccer players may be up to 3 times more likely to sustain an injury since the onset of the EPPP [21], which in turn will reduce a player’s availability to train, improve their sports-specific skill, and potential chances of progression [22]. Opposingly, other work has found a decrease in injury incidence when comparing pre-EPPP to post-EPPP (3.0/1000 h vs. 2.1/1000 h, respectively) [23], highlighting the role different management strategies play in these outcomes. Consequently, sports science and medicine practitioners working in highly-specialised elite development programmes should strive to ensure effective management systems are in place that maximise player availability and potential, reduce the likelihood of injury, and fundamentally ensure a long sporting career and healthy life [19].

**Figure 1:**
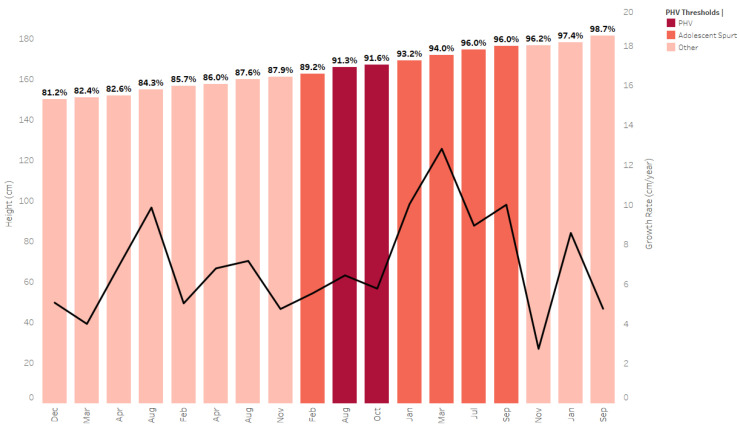
Longitudinal overview of an individual’s growth curves throughout their academy journey. Key: PHV = 90–92% PAH; Adolescent Spurt = 88–96% PAH. PHV: Peak height velocity; PAH: Predicted adult stature. Percentage figures on bars represent % of predicted adult stature; the black line indicates growth rate. Dashboard produced in Tableau Desktop Software (version 2021.1, Salesforce Company, Seattle, WA, USA).

Youth athletes (categorised as children ≤ 13 years and adolescents between 14–18 years [24]) face unique challenges with regards to the inconsistency of the timing and tempo of their physical growth and maturity [25,26]. Pertinently, individuals of the same chronological age can vary in maturity status by as much as 5–6 years in biological age [27]. Importantly, individuals who possess advanced maturity in comparison to their age-matched peers can possess advantages in sporting performance through improvements in aerobic and anaerobic capacities, muscular strength, power, and sprint speed, as a product of pubertal development [25,28]. Growth and maturation are not synonymous, as growth rate is defined as changes in an individual’s body size or parts of the body (e.g., lower limb growth) over time, whereas biological maturation refers to the status, timing, and tempo of progress towards a fully ‘adult’ state (Figure 1) [28,29]. Additionally, ‘maturity status’ can be defined as the state of maturation at the time of observation and ‘maturity timing’ is defined as the age at which a specific maturational event occurs [28,29]. During the pubertal growth spurt, academy soccer players can have growth rates of up to 7.5 to 9.7 cm/year between 10.7 and 15.2 years of age [30,31]. These aforementioned factors are important elements to monitor in youth athlete settings, whereby growth [32,33,34] and maturation [35,36,37] have both been identified as dynamic moderators for potential injury risk.

It is important to have an understanding of the sport’s physical requirements to make informed decisions on training prescription at the individual level [8,38,39,40]. Undoubtedly, the most realistic approach to reveal the game’s demands is via analysis of actual match play [39]. At the senior level, advanced automated analysis systems [38,41,42,43,44,45] allow for the tracking of work rate and activity patterns during match play, through external volume metrics—such as distance covered at various intensities [40,46,47,48], exercise intensity distribution [41,42], indications of fatigue [41,45], and position-specific differences in running performance [42,49]. The tracking of external measures can outline the physical output completed by a player, which can help identify an individual’s physical capacities; however, the utility of internal measures as a means of identifying the relative physiological and psychological stress imposed on an individual is also vital in determining the game requirements and subsequent adaptation imposed by these demands [50]. Thus, the combination of both external and internal volume, intensity, and frequency parameters can highlight the relative physiological and biomechanical demands of soccer for an individual. This will enable the identification of strengths and deficits within a player’s fitness capabilities, which can provide practitioners with a ‘data-informed’ framework from which to develop optimal conditioning strategies for the preparation of the team and the individual [51]. These methods have become commonplace within elite senior soccer team settings and have allowed for the monitoring and periodisation of players’ fitness and fatigue throughout a competitive season to optimise performance levels and mitigate injury risk to improve player availability [52,53,54]. However, the financial, time, and sometimes ethical constraints (e.g., philosophical differences) faced by scientists and coaches working at the junior level can create barriers in their application [55]. Thus, those working with youth athletes require practical, yet scientifically driven, monitoring systems that can be implemented which ensures their athletes are supported during years of rapid growth, maturation, and development.

This review will therefore aim to provide a theoretical and practical reference for sports science and medicine practitioners to help them navigate through the complexities of managing the exercise demands of youth soccer players during their academy journey. It is acknowledged that barriers may exist in the application of best practice; however, through an understanding of growth, maturation, and how these elements interact with the youth soccer player’s relative injury risk, performance capabilities, and welfare, these obstacles can be overcome. Those working in academy soccer clubs should be able to implement foundational, age-appropriate, and progressive training methods to optimise athletic performance, reduce injury risk, and ensure the attainment of future potential.

## 2. Holistic Approach within the Multi-Disciplinary Team

A coordinated and systematic approach to training development and exercise monitoring in youth soccer as a means of guiding decisions on training and match exposure can serve to maximise positive training outcomes (e.g., athletic and technical skill development), player welfare, and minimize negative effects (e.g., illness, injury, overtraining, and drop-out) [56]. With that said, the ability to consistently capture and aggregate all aspects of a developing athlete’s training demands remains extremely difficult in youth sport, which may stifle the effectiveness of the intended monitoring practises [57]. As such, at the forefront of any youth athlete management strategy should be the communication feedback loop between the athlete, coach, and members of the multi-disciplinary team (MDT) [58]. Although there is now a breadth of craft knowledge available to us within members of the MDT (i.e., technical coaching, sports science, physiotherapy, psychology, nutrition, player social care and education, etc.), it is crucial that contributions do not become siloed points of view and instead synergistically contribute to a holistic picture of the youth athlete in question. Furthermore, the MDT should avoid a reactive ‘red-flag’ culture with a risk-averse approach to athlete management (i.e., prevention rather than cure). Instead, it should be the goal of the MDT to strive towards a forward-planning, progression-oriented culture, where appropriate support structures are already in place for identified ‘at risk’ individuals. Subsequently, adjustments to the training programme for these individuals can be planned prospectively, which can reduce their relative risk of injury and subsequently maximise the attainment of athletic potential. A crucial tenet of this approach should be to maximise the exposure and subsequent development of each youth soccer player through a progressive, varied, and bespoke stage-appropriate training programme.

Adolescent soccer players have the capacity to train and improve physical performance in response to training demands imposed in the soccer training environment [59]. They may also be exposed to a heightened injury risk through greater frequencies, volumes, and intensities of training, particularly when coupled with the complexities of growth, maturation, sub-optimal physical conditioning, and reduced movement quality associated with adolescence [25,60,61,62]. It remains a major challenge for those working with athletes to determine the nature and magnitude of specific training stressors required to induce positive responses and balance these stressors with adequate recovery to avoid maladaptation and promote supercompensation [59]. Although increasingly complex models are attempting to expand on our understanding of these causal mechanisms underpinning performance and injury risk [63,64,65,66], the ability to predict such outcomes can still aptly be described as the “Quest for the Holy Grail” within sports science and sports medicine [67]. Thus, the use of and regular monitoring of growth, maturation, performance, and training data within a youth setting should not be used as definitive predictive tools for these aspects, but rather, guide our ongoing decision-making processes that are based on scientific rationale, fundamental training principles, and framed through a performance and training progression mindset [58]. The following sections will detail how these elements can be used to inform decisions on youth athlete training management.

## 3. Physiological Considerations for Injury Risk

As mentioned previously, growth [32,33,34] and maturation [35,36,37] have been highlighted as potential injury risk factors in the adolescent athlete population. Research findings have shown that episodes of rapid growth and the period around peak-height velocity (PHV) are associated with increased injury risk in elite adolescent sports [32,37,68,69,70]. The rapid growth of bone length and mass contrast with the ‘lag’ in the adaptation of the muscles, tendons, and apophyses, which can increase the stress on muscle-tendon junctions [32,37,68,69,70]. This potentially explains the development of traction apophyseal growth-plate injuries, such as Osgood-Schlatter’s disease, in youth athletes [71]. These types of injuries, often classified as ‘overuse’ injuries, are prevalent in adolescent soccer populations [72]. Overuse injuries (e.g., tendinopathies and stress fractures) are defined by the concept of an injury occurring in the absence of a singular, identifiable traumatic cause [73], and can result from the failure of the musculoskeletal system to withstand repetitive, submaximal forces over much longer time frames [74]. In theory, the structural tolerance of growth plates and developing bone may be exceeded if a rapidly growing youth athlete is exposed to excessive or repetitive stresses on their musculoskeletal structures [75]. Furthermore, it has been shown that sports specialisation is an independent risk factor for injury in youth athletic populations [16,17] and the prevalence of Osgood-Schlatter’s disease is 4 times greater in highly-specialised athletes [76]. The high exposures to repetitive patterning of soccer activities (e.g., kicking) typically seen in highly specialised academy soccer players can also lead to morphological maladaptations (i.e., Cam-type deformities and femoral acetabular impingement) [75,77,78]. The sensitivity to these issues can be exacerbated during the stage of skeletal maturation (i.e., typically in boys between 12 and 14 years) when the growth plates are open [77,78,79], coincident with the increases in circulating growth hormone and insulin-like growth factor-1, which heightens the bone’s osteogenic responsiveness to joint loading [80].

The lags in musculoskeletal growth and subsequent changes in body segment inertial parameters (i.e., mass, position of COM, moments of inertia, and radii of gyration) apparent in the growing athlete can lead to compromised neuromuscular control during dynamic activities (e.g., running, cutting, and landing) and is suggested to be a key mechanism for lower-limb ligament injuries [81,82]. A high proportion (20%) of injuries in male youth soccer players are acute traumatic ligament sprains at the ankle and knee [72,83,84]. This may be explained by deficits in active muscular protective mechanisms that are unable to adequately support joint torques during dynamic movements involving deceleration and high forces [85], although a causative link is yet to be substantiated. The compromised neuromuscular control, coupled with the vulnerability of bodily tissues—including musculotendinous junctions, ligament structures, growth cartilage, and bone mineral density—during this period may subject the highly specialised elite youth soccer player to increased injury risk; particularly if movement competency is not maintained, loading variability is limited, and tissue-specific weaknesses are not addressed.

## 4. Elite Training Programme

The training practises of highly-specialised elite youth soccer players are a key component in ensuring players receive the appropriate coaching and training exposure necessary to develop their skillset. Yet it should also be acknowledged that early-specialisation practises have been shown to predispose these individuals to an increased injury risk [70,72]. Therefore, a technical training programme that fails to consider the aspects of growth, maturation, and fundamental training principles may exacerbate these risks, if not addressed through appropriate compensatory programming [15,16]. A highly popular training method in soccer is the use of small-sided games (SSGs), which can be manipulated using various constraints to elicit an intensified physiological stimulus that typically serves as a form of sports-specific high-intensity interval training [86]. Further, pedagogical principles can be integrated to help the youth athlete improve specific technical skills or tactical behaviours aligned to a club’s coaching philosophy [87]. As such, SSGs can provide simultaneous development of physical, technical, and tactical performance qualities [87,88]. However, academy systems may fall guilty of an over-emphasis on small-sided games in order to enhance technical skill development [89]. This may increase the risk of over-use injury through the performance of high and repeated exposure to mechanically demanding activity, repetitive movement patterns, and sports-specific skills [75], as well as under-expose players to the high-speed running demands of match play [89].

Elite academies often partake in seasonal domestic or international tournament matches, whereby condensed fixture schedules require players to perform multiple matches within a few days (typically between 2–4 days). Youth athletes may in fact be more fatigue-resistant than senior athletes [90]; however, the periods of intense competitive match play during these tournaments are reflective of the physical demands placed their elite senior counterparts, who are required to repeat performance in over-intensified competitive schedules with limited recovery opportunities [91]. These experiences certainly represent a challenge and are valuable development opportunities from technical, tactical, physical, and psychological perspectives. Practitioners should also be wary of the reduced loading capabilities of the rapidly growing athlete, which needs to be managed during dense periods of match play to allow sufficient time for rest and recovery for tissue homeostasis [16]. Moreover, disparities in growth and maturity status between individuals performing within the same chronological age groups are often apparent and have been identified as factors that may affect the injury risk [62,92]. This may be explained by the large inter-individual variation in physical and neuro-developmental pathways between players, which brings with them a contrast in morphological structures and neuromuscular control [62,92]. This relationship remains unclear; however, the same way an individual may demonstrate a physical competitive ‘advantage’ by being more mature, their underdeveloped counterpart may demonstrate a physical ‘disadvantage’ which predisposes them to increased risk of sustaining an injury [32,69].

## 5. Developing a Framework for Performance Monitoring and Injury Surveillance

Elite soccer clubs aim to offer an advanced soccer development and education programme, with age groups starting potentially starting from under-5 s through to under-23 s, supported by expert and dedicated staff. A soccer academy high-performance training programme should strive to facilitate an environment where success is inevitable, using every available resource and knowledge base to maximise appropriate player exposure at any age, whilst still mitigating injury risk [93] and promoting player welfare. As such, the goal of the MDT should be to push athletic boundaries and inspire athletes to unlock their true potential [93]. Furthermore, a performance-oriented approach may in fact present as a dual benefit in progressing athletic performance while simultaneously reducing injury risk [94,95,96,97,98,99,100,101]. Resultantly, practitioners should aim to evaluate the effectiveness of their programmes by establishing global and specific key performance indicators (KPIs) [93]. This approach is common in business strategy and facilitates the objective assessment and monitoring of organisational performance relative to its objectives [102], which in this instance would be to improve athletic performance, reduce injuries, and maximise player availability. The use of the following global statistics are therefore suggested for these means and can be presented at both the squad- and individual-level:

1. Match exposure: The total number of soccer match hours or minutes.
2. Training exposure: The total number of training hours or minutes.
3. Match availability: Percentage of the total available team matches against those missed due to injury or illness.
4. Training availability: Percentage of the total available training sessions against those missed due to injury or illness.
5. Injury incidence: Number of injuries per 1000 h of exposure (training, match play, or combined) [103].
6. Injury burden: The total number of days lost to injury per 1000 h of exposure (training, match play, or combined) [103].
7. Bradford factor: Time absent multiplied by the square of the number of injuries [19].

As such, benchmarks can be established for specifically identified KPIs (e.g., strength, power, speed, endurance, movement quality, growth, and maturation) which may be assessed, trained, and monitored cross-sectionally and longitudinally (Table 1 and Table 2) [93]. In addition, with recognition of maturity- and population-specific injury risk patterns, and how these may interact with the elite youth soccer player’s training programme, the development of ‘injury-risk profiles’ can be established as a key initiative for effective performance monitoring and surveillance frameworks for youth athletes [104]. This information will inform more bespoke training strategies that are relative to the individual’s needs. Recent work has shown that youth soccer players may sustain specific injuries at different percentages of their final adult stature [105]. In this instance, growth injuries may appear to follow a chronological distal to proximal pattern, with muscle and joint injuries occurring more frequently in mature players [104,105]. Importantly, although the period of PHV may represent an increased incidence of growth-related injuries [32,37,68,69,70], the high growth rates of the lower extremities pre-PHV—and the trunk post-PHV—increase the susceptibility of these areas to injury without necessarily being in a period of rapid increases in stature [105]. Consequently, estimating and longitudinally monitoring the percentage of player’s predicted adult stature (PAH) may represent a viable basis for which effective maturity-specific injury mitigation programmes can be implemented (Table 2) [105,106,107].

## 6. Longitudinal Training Monitoring

Youth soccer match play is an essential part of a player’s physical development across all stages of development. It is frequently underlined that young athletes should not be treated as ‘miniature adults’ [38,138,139] as they possess markedly different physical and physiological processes that attribute to their soccer performance [139]. These differences are observed in match play, where recent studies have suggested that age and maturation impact match running performance in soccer [137,140,141,142,143,144,145]. As such, the relative physiological demands for young soccer players playing the game within the same age category may be hugely variable within different contexts [8]. These more nuanced elements should be considered when evaluating match performance capabilities at the individual level and efforts should be made to ensure comparisons within chronological age bands are coupled with standardised scores relative to their maturity [115,145]. Importantly, such data can provide age-appropriate insights into the athletic and technical requirements of the game, allowing for bespoke training methods [146] and improvement of long-term training management of an academy player [140], thus avoiding the replication of methods utilised in senior players [8]. Moreover, when considered in absolute terms, this data can also be used to inform when talented young players are physically capable of demonstrating performance outputs that are sufficient to compete when moving up to play in older age brackets [8]. Such information facilitates the effective ‘scaffolding’ of age group requirements around a LTAD framework, allowing practitioners to reverse-engineer their weekly training programmes at the micro- and meso-level to guide developmentally appropriate training prescription.

**Figure 2:**
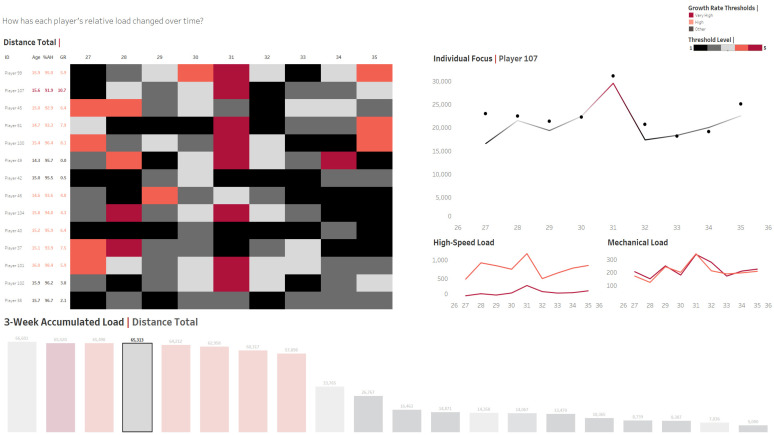
An interactive dashboard which provides a macroscopic overview of external training volume over time—alongside age, maturity status, and growth rate—in a group of academy players. Key: %AH = Percentage of predicted adult stature; GR = Growth rate; 1 to 5 colour thresholds are based on z-scores that represent a players’ normal distribution curve for each external metric (i.e., 15/20/30/20/15th percentiles). Dashboard produced in Tableau Desktop Software (version 2021.1, Salesforce Company, Seattle, WA, USA).

In contrast to the senior game, those working in academy settings are afforded the opportunity to evaluate a training programme with a long-term vision in mind. Therefore, the use of periodisation strategies should permit the structure of a training programme in logical and appropriately sequenced phases and cycles, following specificity and progressive overload principles. With the correct balance of training (i.e., stress) and recovery (i.e., adaptation), training strategies can be used to physically prepare the youth soccer player for the increasing physical demands as they progress towards the senior game [147]. For this management to be effective, an understanding of the longitudinal structure of the adolescent player’s programme is important to define suitable training doses and risk thresholds which can optimise performance and recovery (Figure 2). Limited published information is available with regards to the longitudinal training demands in youth soccer academies (i.e., ≤16 years). To date, studies have been reduced to quantifying youth player match and training demands over one-to-two weeks [11,148], or have solely reported the training durations [108], internal [11], or external demands of training [135]. Collectively, a general trend is reported towards a progressive increase in overall physical loading (i.e., exercise volume) as chronological age increases. More detailed investigations are certainly warranted that examine the chronic physical demands and exercise volumes of academy soccer players from different academy environments with different training philosophies. This detailed insight will enable practitioners to suitably design contextualised training methods which considers the interaction between training, growth, and maturation over longer time frames. It is, therefore, advised that data specific to the individuals in question be collected to inform practitioners in this regard, where possible. This will allow more appropriate physical trajectories to be established, risk thresholds to be identified, and enable long-term training decisions to be informed through a progressive standpoint.

The need to monitor the short- and long-term implications of the internal and external training stressors remain key in longitudinally managing young soccer players. Injury patterns in youth athletes follow a specific aetiology according to their stage of maturation [105], which will have implications for how these stressors interact with the youth athlete at both the acute and chronic level [149]. In addition, the intermittent and multi-directional nature of soccer means the variation in stimuli and response will impart varying degrees of physiological and musculoskeletal demands [61,98,99,150]. For example, neuromuscular fatigue driven by high-speed activity may cycle more transiently within the physiological system [151] and numerous investigations have found associations between acute weekly rapid increases in high-speed running volumes and increased risk of soft tissue injury [109,110,152,153,154]. Conversely, high cumulative totals (i.e., 2- to 4-week accumulative totals) of distance covered [152,155], number of decelerations [152], as well as ‘relative velocity change’ (e.g., algorithmically derived total accelerations, decelerations and change of directions) [155] have been linked to overuse injury. This may be explained by a ‘mechanical fatigue failure’ phenomenon, in which the mechanical fatigue of tissue is perpetuated by accumulated damage as a result of summative and repetitive loading events, subsequently surpassing the remodelling rate of the biological tissue [156]. The heightened responsiveness of the musculoskeletal structures to joint loading in the rapidly growing athlete [80] points towards the importance of monitoring chronic external training volume (Figure 2), alongside individual growth and maturation data (Figure 1), to support informed decisions on youth soccer players during their intensive training programme.

Equally, the evaluation of internal stress should remain a central component within monitoring practises, and the utility of practical, subjective measures allow this data to be readily collected on the youth soccer player [52,157,158,159,160]. Indeed, the psychophysiological response experienced from a specific exercise bout may vary depending on a host of modifiable and non-modifiable contextual factors [161]. The complexities of rapid and varied growth, maturation, and development within a group of adolescent athletes may exacerbate these differences in the response to the prescribed the same training [28]. Furthermore, it should be acknowledged that common indices of training intensity (e.g., session-RPE) may still only provide a ‘global’ evaluation of the internal response and will not detect the mechanical stresses and strains experienced within the muscles, tendons, ligaments, bones, and cartilage (i.e., musculoskeletal tissue). As such, being able to differentiate between the physiological and biomechanical internal response to various external stressors may assist in developing more appropriate and specific loading paradigms in youth soccer [150]. For example, using differential-RPE, a player may be specifically asked to rate their degree of ‘breathlessness’ and ‘leg muscle exertion’ during a session or activity [157,162,163,164], or—through a wellness questionnaire—gage their degree of ‘muscle or joint soreness’ and ‘fatigue’ in the days following a session, and also sleep quality [52,158]. In addition, monitoring symptomology and encouraging the youth athlete to delineate specifically the location of their pain or soreness may deliver further insights [119,165]. This data can be used on the daily level to adjust training or analysed longitudinally to evaluate trends with respect to the external versus internal demands of training, and subsequently, inform periodization strategies. More research is certainly warranted to validate these novel methods, particularly in their applicability for use in youth athletes [163,166]. The harmony of the MDT is pivotal in situations such as these, where roles and responsibilities are clear, expertise is channelled suitably, and athletes are continually educated to maximise the outcome of these measures [93].

## 7. Applying Theory to Practise

Practitioners working in soccer academies should be able to readily apply the tools and theoretical considerations discussed in this article to support their ongoing management processes of the elite soccer player. Regular assessment (e.g., 2–3 months) of anthropometrics (e.g., standing stature, sitting stature, body mass), in addition to parental stature, can support the development of growth and maturity calculations which can be tracked longitudinally [29,149]. Although a detailed discussion on maturity assessment methods is beyond the scope of this current discussion, the authors advocate the use of the Khamis-Roche predicted adult stature method [106,107], and readers are referred to the following texts for more detailed discussions on this topic [28,29,149]. As illustrated in Figure 1, some of an individual’s highest growth rates can occur within the generic PHV thresholds (e.g., 88% to 95% of predicted adult stature) that have been substantiated by previous findings [107] and utilising these growth and maturation indicators can provide valuable insights to inform training. Indeed, recent work [167] has demonstrated a positive linear relationship between smoothed week-to-week changes in total exposure and injury incidence (*p* = 0.001), resulting in a 168% increase in injury likelihood with a 2 SD change in training duration. Furthermore, a positive linear relationship between growth rate and injury incidence (*p* = 0.031), as well as a non-linear relationship between the percentage of predicted adult stature (peak risk occurring at 92%) with injury incidence. In follow-up work, Johnson [168] found that identifying these aforementioned risk factors, and subsequently providing a modified programme, could significantly reduce injury incidence and burden in adolescent soccer players. This adapted training programme involved small adjustments of team-based soccer training volume, which was replaced with low intensity individual sport-specific skills, balance, coordination, impact attenuation, and individualised strength sessions [15,169,170].

As such, by no means do these situations have to become reactive ‘red flag’ scenarios. Instead, when planned for proactively and with appropriate rationale, instances such as these should be seen as an opportunity to provide an individualised stage-specific training programme and progress their athleticism, while reducing the likelihood of injury. Regular assessment of an individual’s athletic profile (e.g., strength, power, speed, flexibility, and movement quality) can determine an individual’s strengths and weaknesses which can inform training prescription. This can be further supported by the numerous performance models [14,15,61,171] and training guidelines that exist within the LTAD area, which can be used to guide athletic development training (e.g., resistance training [24,172,173,174] and multi-directional speed training [61,175,176,177]).

Using evidence-based training programmes which conform to the scientific principles of training, not only can these individuals’ relative injury risk be reduced, but they may also concurrently enhance their biomotor and physical performance capabilities which are fundamental for a successful transition into the senior game [178].

### A Case Study Example

Using the working example presented in Figure 2, a deeper look into ‘Player 107’ was warranted due to their high growth rate (i.e., 7.2 cm/year; “very high”) combined with their current maturity status (i.e., 91.6% AH; “PHV territory”). Notably, the heightened training volume experienced during ‘Week 31’ due to training with an older age group caused the player to reach a high relative loading threshold for several external training volume measures. With acknowledgement of this, alongside the utility of the global (e.g., training availability, Bradford factor, etc.) and specific (e.g., strength, power, and speed profile) KPIs discussed in earlier sections, objective information around the youth athlete can be aggregated, enabling a ‘decision tree’ process to formulate and context to build around an individual’s training status and risk profile. Consequently, conversations between members of the MDT can be implemented, centring around both preparation for the approaching weeks, as well as a potential reduction in soccer-specific training volume during the following weeks, as demonstrated by the individual’s relative ‘3-Week Accumulated Load’ for total distance at ‘Week 33’ [109,167,179]. As a result, a balance may be reached between mitigating the risk of overuse injury while appropriately exposing the player to ‘challenging’ situations, which is key for their continued development.

The resource hierarchy often observed in academy settings means that more advanced monitoring systems (e.g., player tracking technology) tend to be incorporated with older age brackets (e.g., >U15), or an academy with limited resources may not have this facility at all. However, getting the ‘basics’ right, and obtaining simple, accurate, and standardised information on a few research-validated measurement tools can still readily be scaffolded over an entire academy as an effective management system. As mentioned previously, the use of such data within an academy environment should not be solely relied upon, but rather, used to build context around the player and allow subsequent conversations between the MDT to be informed through an objective perspective.

## 8. Conclusions

The information we now have on players is becoming increasingly more applicable to the end-user with the development of new technologies and data visualisation software. For example, there is now the availability for athletes to remotely self-report psychophysiological measures on their devices away from the training facility increasing digital accessibility [159,160]. However, the art of face-to-face communication becomes important as ever in distilling the swathes of information into meaningful messages, in particular to the ‘non-experts’ who are typically at the centre of which all decisions are impacting (i.e., the athlete and technical coach) [58]. Furthermore, the role of the coach and sports scientist in educating their youth athletes on understanding the “why” of these abovementioned strategies is important in achieving their long-term goals [93]. The young athlete needs to build trust in the process, and eventually possess the tools and understanding to become a self-sufficient, resilient individual who thrives in the senior game on their own.

The large disparities in maturation status within academy settings and the growth-related issues, particularly for individuals going through accelerated periods of growth, necessitates an individualised approach to the management of the adolescent soccer player. Furthermore, elite youth soccer players are inherently predisposed to greater injury risk due to their large involvement in highly-specialised sports-specific practice, and so practitioners have a duty of care to provide safe and effective sports science practise for their athletes. Developing a performance monitoring and injury surveillance framework within an academy system can be supported by an understanding of growth, maturation, youth soccer performance, and epidemiology. Subsequently, bespoke programming can be provided which may offset these inherent injury risks elite soccer players are predisposed to, while developing athletic performance. Practitioners should possess a sound theoretical understanding of the training process, and utilise data, where available, to guide ongoing decision-making. Those with limited resources, however, can still use practical methods discussed in this review as evidence-based alternatives when managing their youth athletes. To conclude, a summary list of practical strategies, ordered from simple to more advanced, is provided for consideration:

1. Communication between the MDT and the player—Utilising the extensive resource of the MDT to establish context around an individual while continuing to have ongoing discussions with players to understand their needs.
2. Monitoring exercise activity—Using exercise duration to track exposure and further categorising into specific activity types (e.g., match play, sports-specific, athletic development and extra-curricular activity). Establishing normative values and thresholds for an individual’s activity to determine relative increases or decreases in exposure (e.g., ‘time on feet’).
3. Collection of growth and maturity data—Regular anthropometric assessment (e.g., 2–3 months) to allow for cross-sectional (e.g., maturity status) and longitudinal (e.g., growth curves) evaluation of individuals.
4. Establish athlete profiles, plan and progress athlete training—Evaluate key athletic qualities and establish benchmarks specific to age, maturity, and position. Tracking of specific athletic development exercises (e.g., gym-based and field-based activity) and using published literature as well as previous experience as a guide for age-appropriate training prescription. Using fundamental scientific training principles while using the above points to inform progression or regression of training.
5. Monitoring subjective indices of load—Daily collection of RPE and wellness (e.g., sleep, fatigue, soreness, and stress) to establish values and thresholds for individuals to detect changes. Further classifying scores relative to the physiological (e.g., “breathlessness”) and biomechanical (e.g., “leg exertion”) characteristics to establish more specific load-response profiles.
6. Monitoring of training activity using tracking technology—Establish age-, position-, and maturity-specific match activity profiles and extending this longitudinally to map out a progressive training framework over a LTAD pathway. Monitor chronic exposure to training stressors, understanding the unique physiological and biomechanical load-adaptation pathways underpinning KPIs, and how these may interact with growth and maturation.

## Figures and Tables

**Table 1:** Injury risk profile and potential mitigation strategies for the elite adolescent soccer player with reference to the technical training programme. A periodised, multi-component athletic training programme is advised at all stages of the programme in addition to these specific strategies.

Injury Risk Mechanism	Rationale	Assessment and Monitoring Considerations	Intervention Strategy and Exercise Example (s)
Highly specialised sports-specific technical practise [70,72]	Increase movement diversification and variability to reduce repetitive soccer-specific movement patterns	Monitor soccer-specific, gym-based, multi-sports and extra-curricular training exposure [19,108].	Supplement or replace soccer drill exposure with multi-sports activity (e.g., rugby, basketball, hockey, American football, gymnastics, free running, swimming, tennis, combat sports) [15].
Over-exposure to SSG format technical practise with reduced pitch dimensions [75,89]	Reduce over-exposure to mechanically demanding activity and ensure players are prepared for HSR demands of match play	Monitoring acute and chronic acceleration, deceleration, COD *, and HSR volumes [109]. Quantitative and qualitative assessment of sprinting intensity [110] and technique [96].	Supplement technical training with HSR exposure in athletic development training [89,111]. Ensure weekly attainment of >95% MSS intensities [110].
Large disparities in maturation within chronological age groups [32,62,92]	Reduce variation in maturity status between individuals to balance physicality	Regular growth and maturation assessment (e.g., 2–3 months) [29].	Bio-banded training and match play [112,113,114]. Individualised and stage-appropriate training targets and standards [14,15,115].

Key: SSG = small-sided games; LSG = large-sided games; HSR = high-speed running; COD = change of direction; MSS = maximum sprinting speed. * COD count and distance should be monitored during athletic development training due to current limitations of tracking technologies.

**Table 2:** Injury risk profile and potential mitigation strategies for the elite adolescent soccer player with reference to maturity-based injury risk factors. A periodised, multi-component athletic training programme is advised at all stages of maturation in addition to these specific strategies.

Injury Risk Mechanism	Rationale	Assessment and Monitoring Considerations	Intervention Strategy and Exercise Example (s)
Pre-PHV (<88% PAH)			
Higher incidence of growth-related injuries in extremities (e.g., Sever’s) and ankle joint/ligament injuries [104,105]	Develop foot/ankle strength, neuromuscular control, and localised tissue robustness and mobility.	Movement quality, neuromuscular control (e.g., QASLS [116], LESS [117], TJA [118]). Symptomology [119].	Linear and curvilinear sprint exercises (e.g., sprint races and sprint drills) [61,98]. Jumping/landing competency and impact attenuation training (e.g., plyometrics in various directions, intensities, and surfaces) [120,121]. GSA complex training (e.g., pogo hop variations, end-range isometrics) [121].
Circa-PHV (88–96% PAH)			
Higher proportion of knee joint/ligament injuries [81,105]	Improve movement competency, frontal plane neuromuscular control, and localised tissue robustness.	Movement quality during COD, jump-landing and squatting patterns (e.g., CMAS [122], QASLS [116], LESS [117], TJA [118]). Multi-joint lower-limb maximal and rapid force production (e.g., IMTP, CMJ, RSI, horizontal jumps/hops) [123,124,125,126,127]. Symptomology [119].	MDS competency training (e.g., deceleration and cutting technique) [61,100,101]. Jumping/landing competency and capacity training (e.g., plyometrics in various directions, intensities and surfaces) [120,121].
Higher incidence of growth-related injuries more proximally (e.g., Osgood’s and pelvic avulsions) [104,105]	Improve knee flexor and extensor strength.	Isolated strength qualities during single- and multi-joint actions (e.g., isokinetics, Nordics) [127,128,129]. Symptomology [119].	Compound and isolated RT (e.g., multi-planar SL compound lifts, Nordics) [130,131].
Post-PHV (>96% PAH)			
Higher proportion of groin/spine injuries [104,105]	Develop glute, groin, hip and core strength, and stability.	Multi-joint lower-limb maximal and rapid force production (e.g., IMTP, CMJ, RSI, horizontal jumps/hops) [123,124,125,126,127]. Isolated strength qualities during single- and multi-joint actions (e.g., isokinetics, adductor squeeze, abductor pull) [127,128,129]. Symptomology [119].	Multi-component RT (e.g., heavy DL compound lifts *, glute bridge progressions, Copenhagen exercises). MDS capacity training (e.g., high-intensity deceleration, pivoting and maximal velocity sprinting) [61,98,100,101].
Higher prevalence of muscular injuries [105]	Increase physical capabilities to tolerate the systematic increase in training exposure and physical demands	Quantification of training volume, intensity, frequency, type and response (e.g., GPS, RPE, wellness) [132,133,134].	Establish age, maturity and positional benchmarks for match and training demands [11,108,135,136,137].
Soccer-specific overuse injuries	Avoid repetitive actions and provide variation in soccer-specific activity	Quantification of kicking volume, intensity, frequency and type (e.g., wearable monitoring devices).	Gradual and progressive overload of kicking volumes.

Key: PAH = predicted adult height; LESS = landing error score system; TJA = tuck jump assessment; QASLS = qualitative analysis of single leg squat; IMTP = isometric mid-thigh pull; CMJ = countermovement jump; RSI = reactive strength index; GPS = global positioning system; RPE = rating of perceived exertion; GSA = gastrocnemius-soleus-Achilles; MDS = multi-directional speed; RT = resistance training; SL = single leg; DL = double leg. * Strength training intensities at >85% 1 RM.
