# Correction: McBurnie et al. Training Management of the Elite Adolescent Soccer Player throughout Maturation. *Sports* 2021, *9*, 170

**DOI:** 10.3390/sports10110173

**Published:** 2022-11-03

**Authors:** Alistair J. McBurnie, Thomas Dos’Santos, David Johnson, Edward Leng

**Affiliations:** 1 Football Medicine & Sports Science, Manchester United F.C., AON Training Complex, Manchester M31 4BH, UK; 2 Department of Sport Science, School of Science and Technology, Nottingham Trent University, Nottingham NG1 4FQ, UK; 3 Musculoskeletal Science and Sports Medicine Research Centre, Department of Sport and Exercise Sciences, Manchester Metropolitan University, Manchester M15 6BH, UK; 4 Manchester Institute of Sport, Manchester Metropolitan University, Manchester M1 7EL, UK; 5 Department for Health, University of Bath, Bath BA2 7AY, UK

The authors wish to make the following corrections to this paper [1]:

Remove Figure 3 and update References 167 and 168.

“167. Johnson, D.; Cumming, S.; Bradley, B.; Williams, S. The Influence of Exposure, Growth and Maturation on Injury Risk in Academy Football Players. *J. Sports Sci.* in press.”

“168. Johnson, D.; Cumming, S.; Bradley, B.; Sheree, B.; Williams, S. Can We Reduce Injury Risk during the Growth Spurt? An Iterative Sequence of Prevention in Male Academy Footballers. Ph.D. Thesis, Training Load in Adolescent Footballers, University of Bath, Bath, UK. in press.”

The corrected references appear below:

Figure 3 has been removed.

“167. Johnson, D.M.; Williams, S.; Bradley, B.; Cumming, S. The Interaction between Training Volume, Growth, Maturation, and Injury Risk in Adolescent Football Players. Paper Presented at: Sport Kongress 2020; 30 January 2020. Available online: https://www.researchgate.net/publication/340175460_David_Johnson_Sports_Kongress_Poster_2020 (accessed on 1 September 2021).”

“168. Johnson, D.M.; Williams, S.; Bradley, B.; Cumming, S. Growth, Maturation and Bio-Banding in Football. Paper Presented at: Webinarium Elitprojektet Online; 16 April 2021. Available online: https://www.youtube.com/watch?t=1291&v=wDE8lyYfZ64&feature=youtu.be (accessed on 1 September 2021).”

The authors would like to apologize for any inconvenience caused to the readers by these changes.
